# The Epithelia of the Kidney

**Published:** 1882-06

**Authors:** 


### THE EPITHELIA OF THE KIDNEY.

In the June number of the *New York Medical Journal and Obstetrical Review*, Dr. Henry B. Millard gives an account of his researches in the anatomy of the renal epithelia, and thus sums up the results : 1. The rods discovered by Heidenhain in some varieties of the tubuli uriniferi are part and parcel of a reticulum present within every epithelium. 2. The reticulum, including its elongated rod-like or sheath-like formations, is the living matter proper. 3. The relation of the rods to the rest of the reticulum of an epithelial body varies greatly, the variation probably being due to different stages or degrees of secretion. 4. The reticulum, including the rod-like formations, in the inflammatory process, both in catarrhal and in croupous nephritis, gives rise to a new formation of living matter, which results in the new formation of medullary corpuscles or pus corpuscles. 5. The structureless membrane is lined by flat endothelia lying between it and the basis of the epithelia of the urinary tubules. 6. In nephritis the endothelia become considerably enlarged, and, in catarrhal as well as in croupous nephritis, they line the urinary tubules after the epithelia have been shed or lost ; they surround the cast in croupous nephritis after the epithelia have perished in the formation of the cast.

